# Attenuation of Diabetes-Induced Defects in Cardiac Sarcoplasmic Reticulum and Myofibrils by Propionyl L-Carnitine Therapy

**DOI:** 10.3390/ijms27219512

**Published:** 2026-10-24

**Authors:** Paramjit S. Tappia, Jaideep K. Gill, Vijayan Elimban, Ruchi Tandon, Naranjan S. Dhalla

**Affiliations:** 1 Asper Clinical Research Institute, St. Boniface Hospital, Winnipeg, MB R2H 2A6, Canada; ptappia@sbrc.ca; 2 Department of Food and Human Nutritional Sciences, Faculty of Agricultural and Food Sciences, University of Manitoba, Winnipeg, MB R3T 2N2, Canada; 3 Institute of Cardiovascular Sciences, St. Boniface Hospital Albrechtsen Research Centre, Winnipeg, MB R2H 2A6, Canadavelimban@sbrc.ca (V.E.); 4 Department of Physiology and Pathophysiology, Max Rady College of Medicine, University of Manitoba, Winnipeg, MB R3E 0J9, Canada; 5 Center for Drug Discovery, BRIC- Translational Health Science and Technology Institute, Faridabad 121001, India

**Keywords:** diabetic cardiomyopathy, propionyl L-carnitine, palmitoyl L-carnitine, sarcoplasmic reticulum Ca^2+^-uptake, sarcoplasmic reticulum Ca^2+^-stimulated ATPase, myofibril Ca^2+^-stimulated ATPase, myosin Ca^2+^-ATPase

## Abstract

Our previous work showed that treatment of diabetic rats with propionyl L-carnitine (PPLC), a metabolic intervention, improved cardiac function and depressed the occurrence of Ca^2+^-overload in addition to attenuating sarcolemmal defects in Na^+^-K^+^ ATPase, Na^+^-Ca^2+^ exchange and Ca^2+^/Mg^2+^-ecto ATPase activities. This study was undertaken to examine the efficacy of PPLC treatment on changes in sarcoplasmic reticulum (SR) Ca^2+^-uptake and Ca^2+^-stimulated ATPase as well as myofibril (MF) Ca^2+^-ATPase activities by employing male Sprague-Dawley rats injected with streptozotocin (65 mg/kg) for the induction of diabetes. Three days after inducing diabetes, diabetic rats were treated with or without PPLC (250 mg/kg/day) for 8 weeks. PPLC treatment was observed to improve cardiac function and attenuate depressions in both SR Ca^2+^-uptake and Ca^2+^-pump ATPase activities without affecting depressions in MF Ca^2+^-stimulated or Mg^2+^-ATPase activities as well as myosin Ca^2+^- or actin-stimulated ATPase activities in diabetic hearts. PPLC treatment also reduced the increased content of cardiac long-chain acylcarnitine as well as the plasma levels of free fatty acids and triglycerides without affecting the elevated levels of plasma glucose or depressed levels of plasma insulin in diabetic animals. The depression in cardiac carnitine content was corrected by PPLC treatment. Under in vitro conditions, palmitoyl L-carnitine (PMLC; a long-chain acylcarnitine) did not affect MF Ca^2+^-stimulated ATPase in the absence or presence of PPLC, whereas the inhibitory effect of PMLC on SR Ca^2+^-stimulated ATPase activities was antagonized by PPLC. These data suggest that the beneficial effects of PPLC in diabetic cardiomyopathy may be associated with attenuation of SR Ca^2+^-handling defects rather than with any action regarding depressed MF ATPase activity.

## 1. Introduction

It is now well known that long-chain fatty acids are the preferred substrate in the heart for the production of energy by mitochondria [1,2]. However, due to the impermeable nature of the inner mitochondrial membrane to long-chain fatty acids, L-carnitine is required as a co-factor for their transport into mitochondria as well as the complete oxidation of fatty acids [3,4]. It is noteworthy that several derivatives of L-carnitine, such as propionyl L-carnitine (PPLC), acetyl L-carnitine and palmitoyl L-carnitine (PMLC), are formed due to the activation of acyltransferase in diseased hearts [5,6,7]. Furthermore, short-chain fatty acid derivatives (PPLC being most active) have been shown to exert beneficial effects in heart disease, whereas long-chain fatty acid derivatives such as PMLC are known to exert toxic effects on the heart [5,6,8]. In fact, deficiency of L-carnitine in heart disease has been shown to be associated with accumulation of PMLC, alteration of action in mitochondrial metabolism, and myocardial cell damage [9,10,11,12,13], whereas therapeutic treatment of chronic diabetes with PPLC was reported to improve the oxidation of palmitic acid and increase the production of ATP in the heart [14]. Both L-carnitine and PPLC have been demonstrated to exert beneficial effects in stimulating fatty acid metabolism in various cardiovascular diseases, such as ischemic heart disease [15,16,17,18,19,20,21,22], cardiac hypertrophy and heart failure [23,24,25,26,27,28,29]. These observations support the view that PPLC is a suitable metabolic therapy for cardiovascular disease for improving cardiac function and the energy status of the heart under different pathological conditions associated with L-carnitine deficiency [30,31,32,33,34,35,36,37,38].

Several studies have revealed that chronic diabetes due to insulin deficiency or insulin resistance is associated with cardiomyopathy and cardiac dysfunction as well as elevated levels of plasma glucose, free fatty acids, triglycerides and cholesterol [39,40,41,42,43,44,45,46]. Although the exact mechanisms for the occurrence of diabetic cardiomyopathy are not clearly understood, developments of oxidative stress, inflammation, mitochondrial Ca^2+^-overload and metabolic defects in the heart have been suggested to be involved in the pathogenesis of cardiac dysfunction in chronic diabetes [46,47,48,49,50,51,52]. Particularly, the remodeling of cardiac sarcolemma (SL) has been shown to be associated with the occurrence of intracellular Ca^2+^-overload, and that of the sarcoplasmic reticulum (SR) results in Ca^2+^-handling abnormalities, whereas defects in both mitochondria and myofibrils (MFs) are associated with alterations in energy production and utilization in the diabetic heart [46,47,51,52,53]. In fact, a wide variety of pharmacological agents, including anti-hyperglycemic agents, antioxidants, Ca^2+^-antagonists, β-adrenoceptor antagonists and blockers of the renin-angiotensin system, have been shown to partially improve cardiac function in diabetic cardiomyopathy by attenuating subcellular remodeling [44,48,52,53]. Various lipid-lowering metabolic agents have also been reported to produce beneficial effects in the diabetic heart by attenuating Ca^2+^-handling abnormalities, inducing a shift from the oxidation of free fatty acids to that of glucose as well as improving cardiac function [54,55,56,57]. Thus, it appears that impairment of cardiac function in diabetic cardiomyopathy may be due to complex mechanisms involving not only metabolic defects but also subcellular Ca^2+^-handling alterations. It should be mentioned that all these observations were made by employing the streptozotocin-induced rat model of diabetic cardiomyopathy, which closely resembles Type 1 diabetes in clinical conditions. However, these observations cannot be generalized to cardiomyopathy in Type II diabetes in humans.

Treatment of diabetic animals with PPLC has been demonstrated to attenuate cardiac dysfunction upon increasing L-carnitine content, improving lipid metabolism and lowering plasma lipids [58]. Furthermore, mitochondrial function was improved, ATP production was stimulated and ATP content was increased in the diabetic heart by PPLC treatment [14,59,60]. Improvement of cardiac function in diabetic cardiomyopathy by PPLC therapy was also demonstrated to be associated with attenuating depressions in sarcolemmal Na^+^-K^+^ ATPase and Na^+^-Ca^2+^ exchange activities, without affecting depression in sarcolemmal Ca^2+^-stimulated activities [60,61]. Previous studies in our laboratory have shown that depressions in the SR Ca^2+^-transport system involving Ca^2+^- uptake and Ca^2+^- stimulated ATPase activities, unlike MF Ca^2+^-stimulated ATPase activity, in diabetic cardiomyopathy were partially prevented by PPLC treatment [60,62]. However, the Ca^2+^-related activities of both SR and MFs in these studies [60,62] were determined at one incubation time by employing only one concentration of Ca^2+^ [60,62]. This study, therefore, was undertaken to examine if the treatment of diabetic rats with PPLC improves SR Ca^2+^-uptake and Ca^2+^-stimulated ATPase as well as MF Ca^2+^-stimulated and MF Mg^2+^ ATPase activities when determined at different incubation times and at different concentrations of Ca^2+^. Furthermore, SR ATP-independent and ATP-dependent Ca^2+^-binding activities as well as myosin Ca^2+^-ATPase and actin-stimulated ATPase activities were examined to establish if the beneficial effects of PPLC in diabetic cardiomyopathy are limited to improving SR Ca^2+^-transport and are not dependent upon changes in MF proteins. The effects of PPLC treatment on myocardial carnitine and long-chain acylcarnitine content were also examined to determine if changes in these parameters are associated with the improvement of cardiac function and SR Ca^2+^-transport activities in the diabetic heart. In addition, this study examined the interaction of PPLC and PMLC (a long-chain acylcarnitine derivative) under in vitro conditions to establish any direct antagonistic action of these carnitine metabolites on SR Ca^2+^-stimulated ATPase and MF Ca^2+^-stimulated ATPase activities.

## 2. Results

### 2.1. Hemodynamic and Metabolic Characteristics

**Figure 1:**
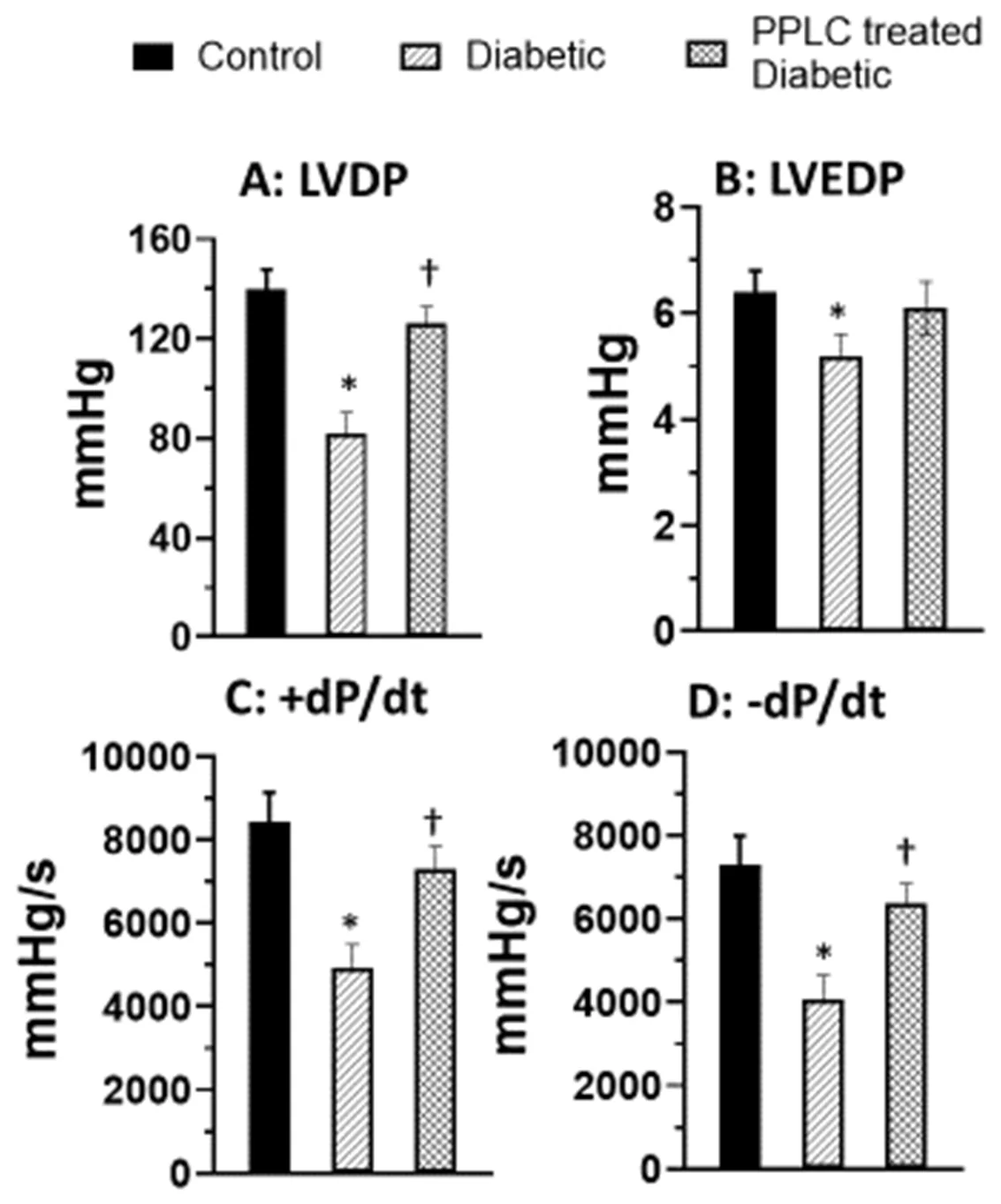
Effect of propionyl L-carnitine (PPLC) treatment on cardiac function in diabetic rats. Three days after the induction of diabetes by a streptozotocin injection (65 mg/kg), diabetic rats were treated with or without PPLC (250 mg/kg/day) for 8 weeks. (**A**) LVDP, left ventricular developed pressure; (**B**) LVEDP, left ventricular end diastolic pressure; (**C**) +dP/dt, rate of increase in developed pressure; (**D**) −dP/dt, rate of decline in developed pressure. Values are mean ± SE of 4 animals in each group. * *p* < 0.05 vs. control group; † *p* < 0.05 vs. diabetic group.

**Figure 2:**
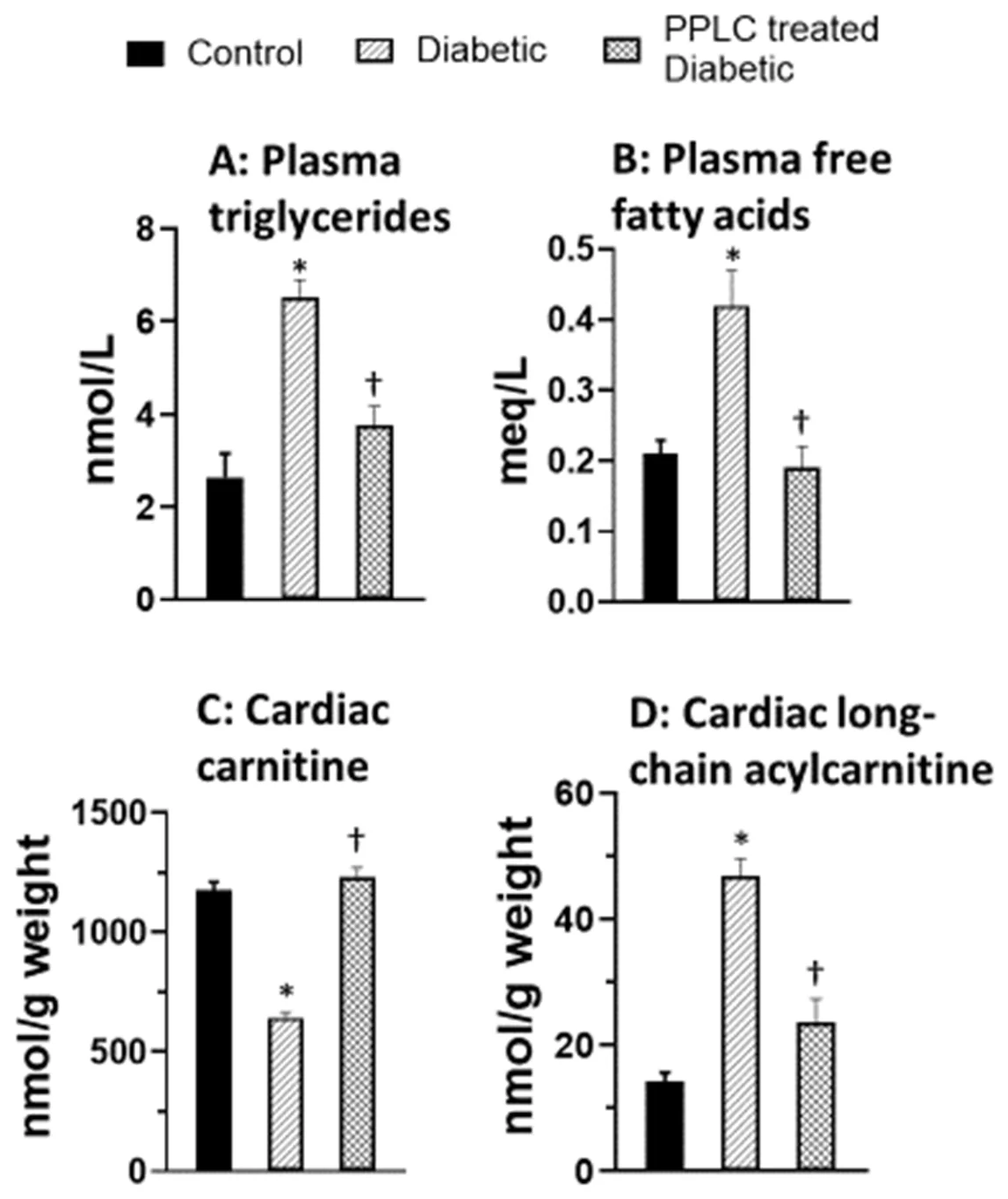
Effect of propionyl L-carnitine (PPLC) treatment on plasma triglyceride and free fatty acid levels as well as cardiac carnitine and long-chain acylcarnitine content in diabetic rats. Values are mean ± SE of 4 animals in each group. * *p* < 0.05 vs. control group; † *p* < 0.05 vs. diabetic group.

Some hemodynamic and metabolic parameters in control and diabetic rats with or without PPLC (250 mg/kg/day) treatment for 8 weeks were measured, and the data are shown in Figure 1 and Figure 2 as well as in Table 1. The cardiac function (as determined from values for LVDP, +dP/dt and −dP/dt) in diabetic animals was depressed and was partially improved upon treatment with PPLC (Figure 1). While LVEDP was slightly decreased in diabetic rats, there was no difference between the values for untreated and PPLC-treated animals. It can be seen from Table 1 that arterial blood pressure and the plasma levels for glucose and cholesterol were increased in the diabetic group, but only blood pressure, unlike the increased values for plasma glucose and cholesterol, was lowered significantly in diabetic animals by PPLC treatment. On the other hand, depressions in heart rate and plasma insulin levels as well as body weight and ventricular weight of the diabetic animals were unaffected by PPLC treatment (Table 1). The data in Table 1 also indicate that the ratio of ventricular weight to body weight was significantly increased in the diabetic group with or without PPLC treatment in comparison to the control animals. The results in Figure 2 indicate that plasma levels of triglycerides and free fatty acids as well as the cardiac long-chain acylcarnitine content were increased, whereas cardiac carnitine content was decreased due to diabetes. All these alterations in metabolic parameters were partially or fully prevented by PPLC treatment (Figure 2). It is also pointed out that treatment of the control animals with PPLC showed no effect on cardiac function, as values for LVDP and LVEDP (mmHg) for the four control animals were 128 ± 7.6 and 6.4 ± 0.05 and those for the four PPLC-treated animals were 125 ± 4.1 and 7.1 ± 0.06, respectively. Preliminary studies in our laboratory using 50 mg, 100 mg, 150 mg and 250 mg PPLC/kg/day for 8 weeks also showed that 250 mg/kg/day was the most effective dose of PPLC in improving cardiac function in diabetic rats.

### 2.2. SR Ca^2+^-Uptake and Ca^2+^-Pump ATPase Activities

**Figure 3:**
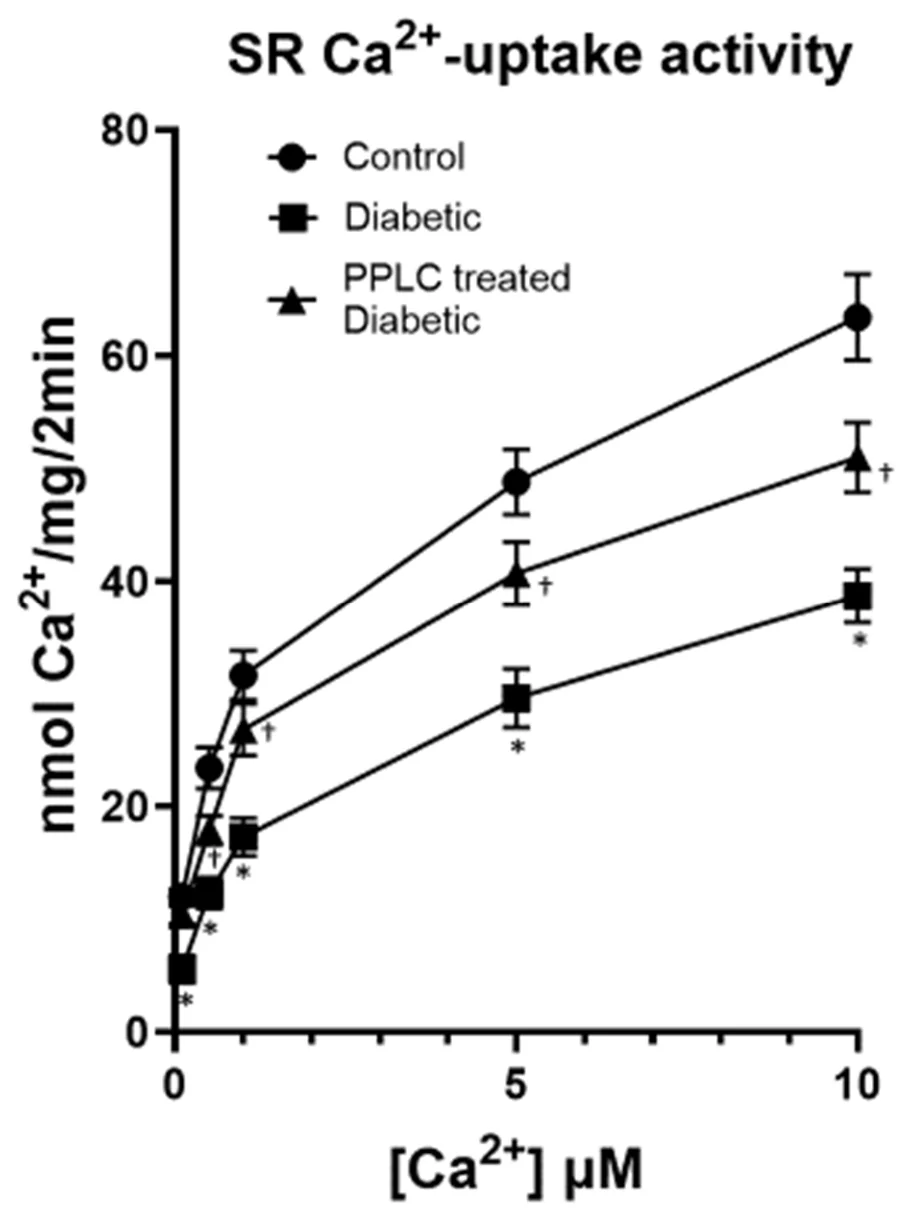
Effect of propionyl L-carnitine (PPLC) on cardiac sarcoplasmic reticulum (SR) Ca^2+^-uptake activity in diabetic rats. SR was isolated from the ventricular tissue from 2 hearts for each preparation. ATP dependent Ca^2+^-uptake was determined in the presence of different concentrations of Ca^2+^. Values are mean ± SE of 4 preparations in each group. * *p* < 0.05 vs. control group; † *p* < 0.05 vs. diabetic group.

In order to gain some information regarding the effectiveness of PPLC therapy on changes in SR Ca^2+^-transport activities in diabetic animals, both Ca^2+^-uptake and Ca^2+^-stimulated ATPase activities were monitored in cardiac SR preparations from control, diabetic and PPLC-treated diabetic animals. The data in Table 2 indicate that the Ca^2+^-uptake activity in diabetes was markedly depressed at different intervals (1 to 20 min) of incubating the SR preparations. Furthermore, the diabetic SR Ca^2+^-uptake activity was depressed when the measurements were made in the presence of different concentrations (0.1 to 10 μM) of Ca^2+^ in the incubation medium (Figure 3). It can also be seen from Table 2 and Figure 3 that treatment of diabetic animals with PPLC prevented the depression in SR Ca^2+^-uptake activity under different experimental conditions. In addition, ATP-dependent Ca^2+^-binding with diabetic SR preparations was also depressed, and this change was partially prevented upon treatment of diabetic animals with PPLC (Table 3). On the other hand, there were no differences in the ATP-independent Ca^2+^- binding with SR preparations in control, diabetic and treated diabetic hearts (Table 3).

**Figure 4:**
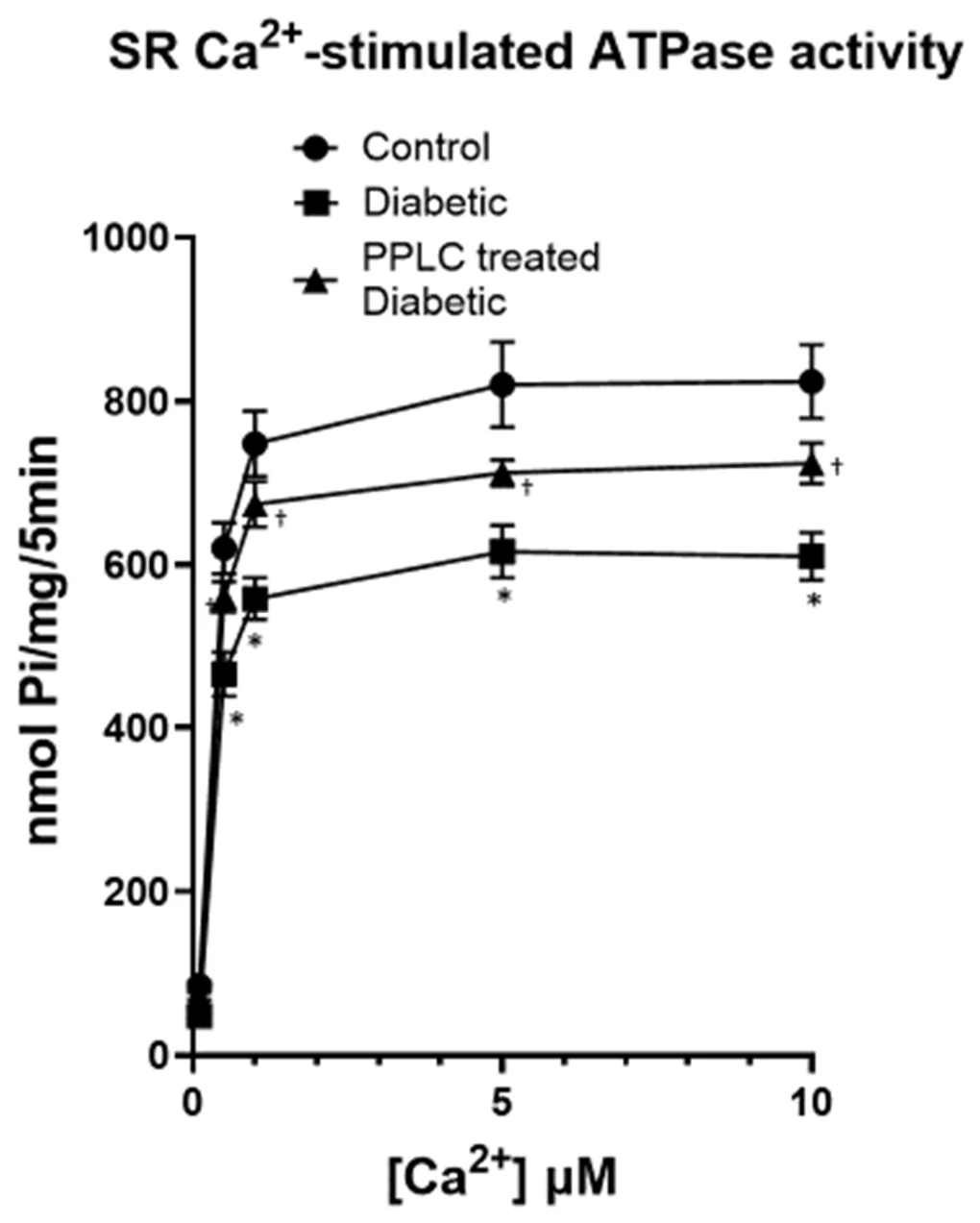
Effect of propionyl L-carnitine (PPLC) treatment on cardiac sarcoplasmic reticulum (SR) Ca^2+^-pump ATPase activity in diabetic rats. SR was isolated from the ventricular tissue from 2 hearts for each preparation. SR Ca^2+^-stimulated ATPase activity was measured in the presence of different concentrations of Ca^2+^. Values are mean ± SE of 4 preparations in each group. * *p* < 0.05 vs. control group; † *p* < 0.05 vs. diabetic group.

In view of the involvement of Ca^2+^-stimulated ATPase in SR Ca^2+^-uptake, SR Mg^2+^-ATPase and Ca^2+^-stimulated ATPase activities in SR preparations from control, diabetic and PPLC-treated diabetic animals were determined, and the results are shown in Table 3. Unlike SR Mg^2+^-ATPase activity, the Ca^2+^-stimulated ATPase activity of SR preparations from diabetic animals was markedly decreased, and this change was prevented upon treatment of diabetic animals with PPLC (Table 3). Furthermore, depression in SR Ca^2+^-stimulated ATPase activity in the diabetic heart and partial prevention of this change in diabetic SR upon treatment with PPLC were also evident, when the ATPase activities were determined in the presence of different concentrations of Ca^2+^ in the incubation medium (Figure 4).

### 2.3. MF and Myosin ATPase Activities

**Figure 5:**
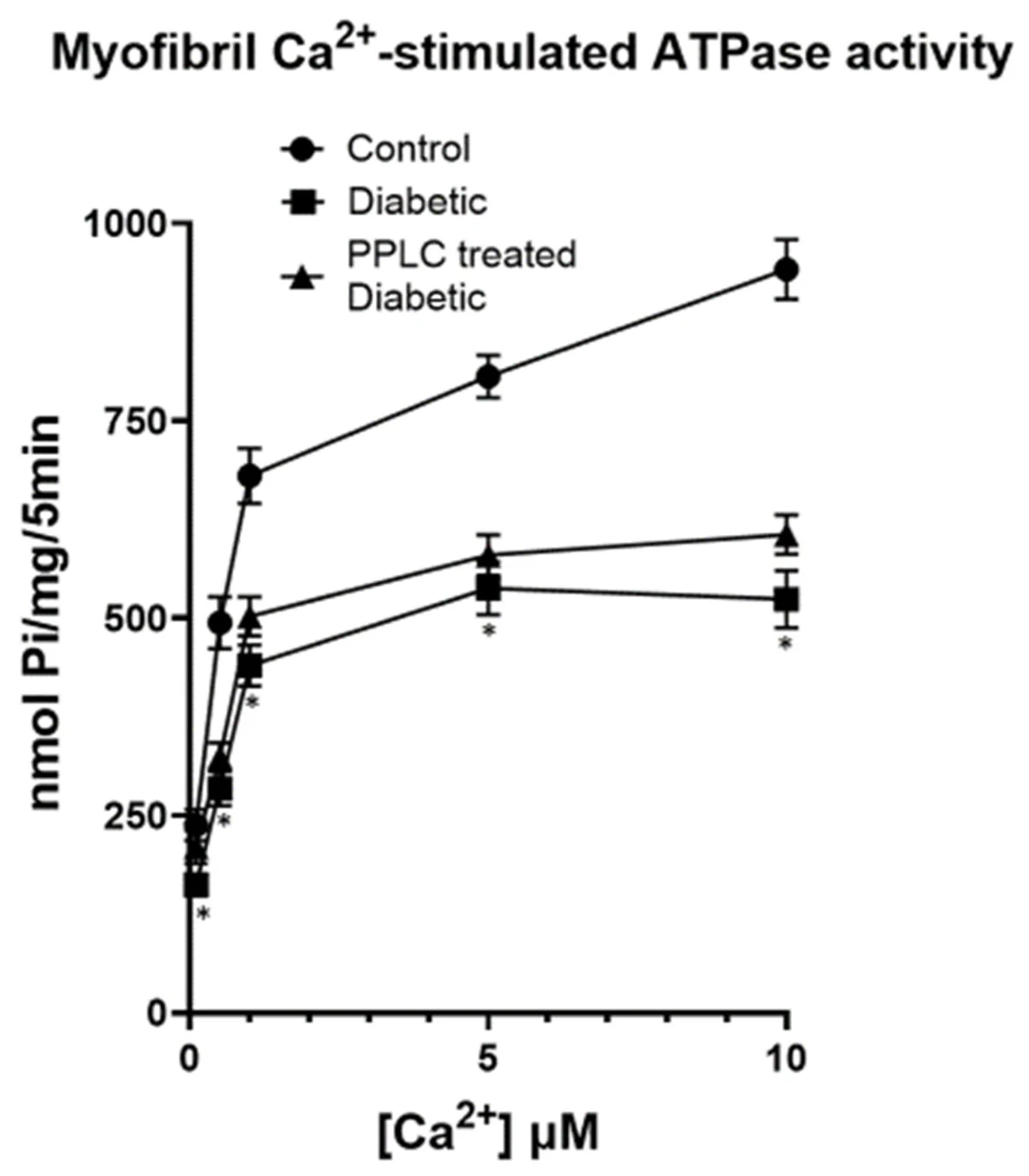
Effect of propionyl L-carnitine (PPLC) treatment on cardiac myofibril (MF) Ca^2+^-stimulated ATPase activity in diabetic rats. Ventricular tissue from 2 hearts was used for each MF preparation. MF Ca^2+^-stimulated ATPase activity was measured in the presence of different concentrations of Ca^2+^. Values are mean ± SE of 4 preparations in each group. * *p* < 0.05 vs. control group.

In order to examine the effects of PPLC treatment on diabetic-induced changes in MF, ATPase activities of MFs and myosin were examined in control, diabetic and PPLC-treated diabetic hearts, and the results are shown in Table 4 and Table 5 as well as Figure 5. It was observed that not only MF Ca^2+^-stimulated ATPase activity was depressed in chronic diabetes, but MF Mg^2+^-ATPase activity was also decreased in diabetic cardiomyopathy (Table 4). Similarly, myosin Ca^2+^-ATPase activity and actin-stimulated ATPase activity were depressed in chronic diabetes (Table 4). However, neither depressed MF ATPase activities nor depressed myosin ATPase activities in the diabetic heart were affected by PPLC treatment (Table 4). It is also noteworthy that depression in the MF Ca^2+^-stimulated ATPase activity in diabetic hearts was also observed at different time intervals of incubation (1–20 min) as well as in the presence of different concentrations of Ca^2+^ (0.1 to 10 μM) in the incubation medium (Table 5 and Figure 5). The depressed MF Ca^2+^-stimulated ATPase activity in diabetic hearts was not affected by the treatment of diabetic animals with PPLC (Table 5 and Figure 5).

### 2.4. Interaction of PPLC and PMLC

**Figure 6:**
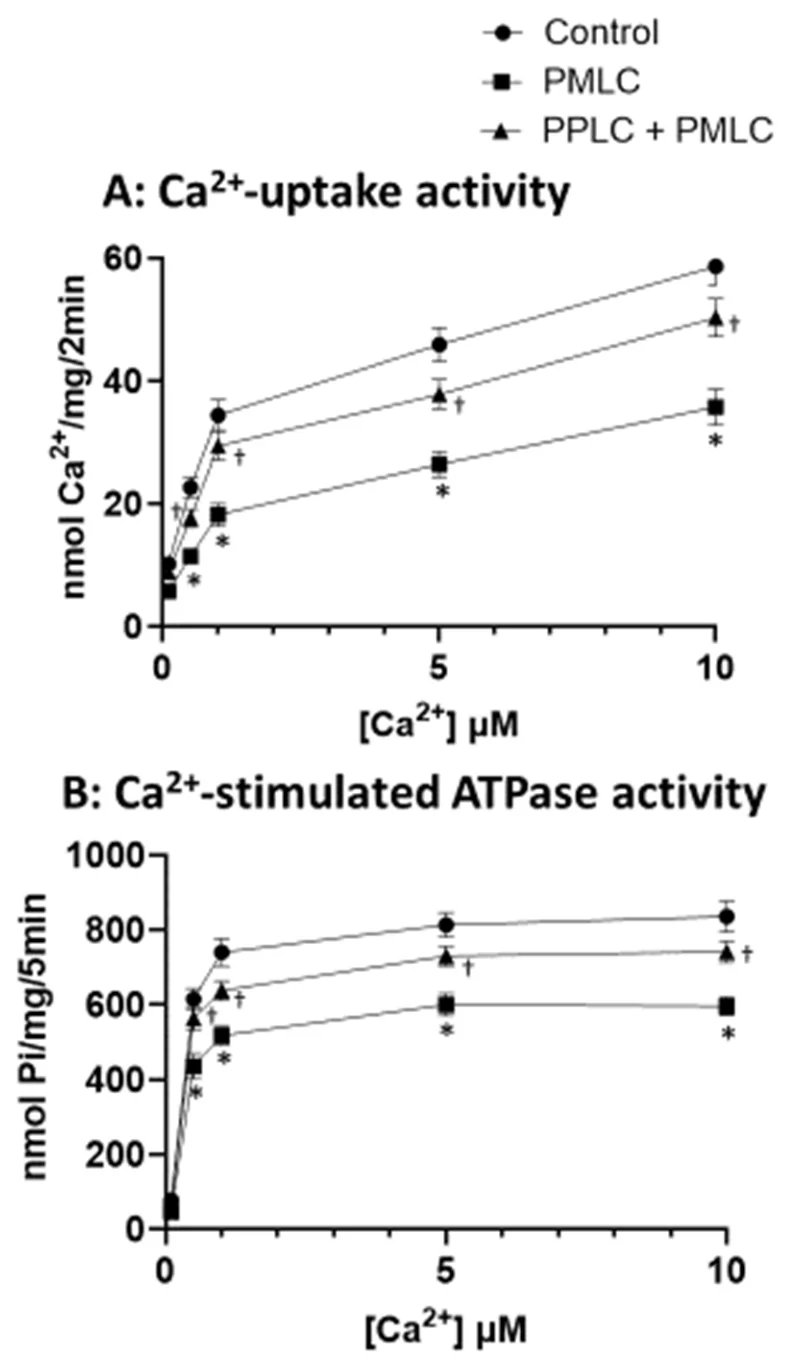
Modification of 50 μM palmitoyl L-carnitine (PMLC)-induced changes in control rat heart sarcoplasmic reticulum (SR) Ca^2+^-uptake and Ca^2+^-stimulated ATPase activities by 50 μM propionyl L-carnitine (PPLC) in vitro. SR was isolated from the ventricular muscle from 2 hearts for each preparation. Values are mean ± SE of 4 preparations in each group. * *p* < 0.05 vs. control group; † *p* < 0.05 vs. PMLC group.

In order to gain information regarding the antagonistic effects of PPLC and PMLC (a long-chain acylcarnitine), we examined the effects of PMLC on control rat heart SR and MF preparations in the absence and presence of PPLC. Incubation of SR with PMLC was observed to depress both Ca^2+^-uptake and Ca^2+^-stimulated activities in the presence of different concentrations of Ca^2+^ (Figure 6A,B). However, pre-incubation of SR membranes with PPLC was observed to antagonize the depressant effects of PMLC on both Ca^2+^-uptake and Ca^2+^-stimulated ATPase activities (Figure 6). On the other hand, incubation of MF with PMLC in the absence or presence of PPLC showed no effect on MF Ca^2+^-stimulated ATPase activity in the presence of different concentrations of Ca^2+^ (Table 6).

## 3. Discussion

In this study, chronic diabetes was associated with depressed cardiac function and heart rate, but arterial systolic blood pressure was increased. Furthermore, body weight and ventricle weight were decreased, but the ratio of ventricle weight to body weight was increased, indicating an increase in the relative cardiac mass in diabetic animals. These observations are consistent with those reported earlier from our laboratory [53,61]. However, other investigators have reported that blood pressure in chronic diabetes is either decreased or unaltered [63,64,65]. Although it is difficult to reconcile the elevated blood pressure in our experimental model of diabetes, the possibility of the involvement of increased levels of several vasoactive hormones [47,48,52,53] in streptozotocin-induced diabetes cannot be ruled out. Nonetheless, this study has shown that depressed cardiac function in diabetic animals was associated with decreased carnitine content and increased long-chain acylcarnitine content of the diabetic heart, indicating that increased levels of long-chain acylcarnitine may play a role in the development of cardiac dysfunction in chronic diabetes. In this regard, it should be noted that increased levels of long-chain acylcarnitine content have also been reported in diabetic and ischemic hearts [66,67]. Increased levels of long-chain acylcarnitine in SR enriched fraction were also observed in diabetic hearts [68]. Furthermore, PMLC, which is the most abundant component of long-chain carnitines, is an inhibitor of SR Ca^2+^-uptake and Ca^2+^-stimulated ATPase activities [68,69,70,71]. These observations support the view that cardiac dysfunction in diabetic hearts may be a consequence of SR Ca^2+^-handling defects due to increased levels of long-chain acylcarnitine [68].

The results show that the development of cardiomyopathy in chronic diabetes is associated with cardiac dysfunction and depressions in SR Ca^2+^-uptake and Ca^2+^-pump ATPase activities as well as MF Ca^2+^-stimulated ATPase and myosin Ca^2+^-ATPase activities. These observations are in agreement with previous studies indicating defects in cardiac function as well as SR Ca^2+^-transport and contractile protein activities in diabetic hearts [47,48,52,53,63,64,65,66]. Since these alterations in cardiac function and subcellular abnormalities were partially prevented upon treatment with a wide variety of agents acting through different mechanisms, it is suggested that cardiac dysfunction and subcellular defects in diabetic cardiomyopathy are elicited through a complex set of pathophysiological factors, such as hormonal imbalance, oxidative stress, inflammation and Ca^2+^-handling abnormalities [44,48,52,53]. The observations that SR Ca^2+^-uptake and Ca^2+^-stimulated ATPase as well as MF Ca^2+^-stimulated ATPase activities determined at various concentrations of Ca^2+^ and at different incubation times not only confirm but also extend our previous findings in this regard [60,62]. The data in this study also indicate that treatment of diabetic animals with PPLC partially prevented depressions in SR Ca^2+^-uptake and Ca^2+^-stimulated ATPase activities without affecting the alterations in MF Mg^2+^-ATPase and Ca^2+^-stimulated ATPase activities. It is pointed out that depressed myosin Ca^2+^-ATPase and myosin actin-stimulated ATPase activities in diabetic hearts were also not affected by PPLC therapy. Such observations indicate that the beneficial effects of PPLC may be related to the prevention of changes in SR Ca^2+^-transport activities rather than due to the prevention of alterations in the contractile proteins. Although our experimental findings demonstrate that the improvement of cardiac function in chronic diabetes with PPLC therapy is associated with attenuation of SR Ca^2+^-handling defects, some caution should be exercised because we did not determine the myocardial content of PPLC in this study.

It is also noteworthy that the cardiac carnitine content was decreased and the cardiac long-chain acylcarnitine content was increased in the diabetic animals. Furthermore, the elevated cardiac content of long-chain acylcarnitine, which inhibits SR Ca^2+^-transport activities without affecting MF ATPase activity, was decreased in the diabetic heart upon treatment with PPLC. Increased levels of long-chain acylcarnitine and depressed levels of carnitine have also been shown by other investigators in the diabetic and ischemic myocardium [66,68,69,70]. It is thus possible that a depression in long-chain acylcarnitine may improve the function of the SR Ca^2+^-transport system in PPLC-treated diabetic hearts. In addition, the improvement of SR Ca^2+^-uptake and Ca^2+^-pump activities in diabetic hearts by PPLC treatment may also be a consequence of the antagonistic effect of PPLC and PMLC. In this regard, we demonstrated that PPLC in vitro reduced the effect of PMLC on the SR Ca^2+^-transport system, unlike MF ATPase activity. Since we measured total long-chain acylcarnitine and not PMLC in the heart, as well as in view of the concentrations of PPLC and PMLC used in the in vitro experiments, some caution should be exercised regarding the antagonistic effect of PPLC and PMLC under in vivo conditions. Nonetheless, PPLC in vitro has been shown to antagonize the depressant effect of PMLC on sarcolemmal Na^+^-K^+^ ATPase and Na^+^-Ca^2+^ exchange activities [61].

Since PPLC has been reported to reduce the elevated level of various parameters for oxidative stress in diabetic cardiomyopathy [61], the possibility of involvement of reduction in the level of oxidative stress in improving SR Ca^2+^-transport activities in diabetic hearts due to PPLC treatment cannot be overlooked. Since alterations in heart rate and blood pressure in diabetic animals are attenuated by PPLC therapy, the improvement of cardiac contractile function due to the contribution of hemodynamic changes cannot be ruled out. Furthermore, it is likely that an increase in SR Ca^2+^-transport activity in diabetic cardiomyopathy by PPLC treatment may also be possible as a consequence of improving the energy status of the diabetic heart [60]. Accordingly, it is suggested that PPLC therapy may improve cardiac function by promoting SR Ca^2+^-transport mechanisms through some lipid-dependent complex mechanisms. On the other hand, the ineffectiveness of PPLC therapy to attenuate depressions in MF Ca^2+^-stimulated ATPase or myosin ATPase activity may be attributed to changes in myosin isoforms in the diabetic heart [72,73]. However, this explanation is unlikely because PMLC-induced depression in MF Ca^2+^-stimulated ATPase was not affected by PPLC under in vitro conditions.

## 4. Materials and Methods

### 4.1. Experimental Model of Diabetes

Male Sprague-Dawley rats weighing about 200 g each were injected with 65 mg/kg body wt streptozotocin (Sigma-Aldrich, Oakville, ON, Canada) for the induction of diabetes. Diabetic state in these animals was verified upon measuring glucose levels at 3 days after injecting streptozotocin by using the tail vein prick and glucometer techniques. Animals showing glucose levels in the range from 440 to 565 (mg/100 mL) were selected for the diabetic group. Rats injected with buffered (0.1 M citrate, pH 4.5) vehicle served as controls. Three days after the induction of diabetes, the diabetic animals were treated with or without PPLC (250 mg/kg/day; subcutaneously) for 8 weeks. The control, untreated diabetic, and treated diabetic animals were sacrificed by decapitation, and the ventricular tissue was dissected out and used for the isolation of subcellular organelles. The ventricular tissue was also stored at −20 °C for biochemical analysis. Blood was collected in heparinized tubes at the time of animal death. Plasma was prepared from the blood sample and stored at −20 °C for biochemical analysis. Methods for the induction of diabetic cardiomyopathy as well as the preparation of other materials are similar to those described elsewhere [61,74,75].

It is pointed out that all experimental animals were housed under appropriate environmental enrichment in the St. Boniface Hospital Research Centre Animal Care Facility. This study used 238 rats (78 control, 80 diabetic and 80 PPLC-treated diabetic). These animals were divided into three groups by the technical staff of the Animal Care Facility. The principal investigator and two data analysts were not aware of the animal group allocation.

### 4.2. Hemodynamic Assessment of Animals

Rats were anesthetized with ketamine (90 mg/kg) plus xylazine (10 mg/kg), and cardiac performance was measured by inserting a mikro-tip pressure transducer (model SPR-249, Millar Instruments, Houston, TX, USA) into the left ventricle. Heart rate, LVDP, LVEDP, +dP/dt and −dP/dt were recorded. The arterial systolic blood pressure was recorded in the right carotid artery, through which the mikro-tip transducer was inserted and then advanced into the left ventricle. The methods for these measurements were the same as used in our laboratory [53,61].

### 4.3. Isolation of SR and Determination of Ca^2+^-Transport Activities

Cardiac SR was isolated from ventricular tissues for each experiment according to the methods used earlier [53,74]. Both Ca^2+^-uptake and Ca^2+^-stimulated ATPase activities were determined in the presence of 10 μM Ca^2+^ (unless described in the text) using methods described elsewhere [53,74].

### 4.4. Isolation of MFs and Myosin and Determination of ATPase Activities

MFs were isolated from 2 to 3 ventricular tissues for each experiment, according to the method described previously [53,75]. Both MF Mg^2+^-ATPase and Ca^2+^-stimulated ATPase activities were determined as previously described [53,75]. Ca^2+^-stimulated ATPase was determined by using 10 μM Ca^2+^ (unless indicated in the text). Myosin was isolated, and Ca^2+^-ATPase as well as actin-stimulated activities were determined by the methods described elsewhere [72,73].

### 4.5. Interaction of PPLC with PMLC for SR and MF Activities

Since SR Ca^2+^-uptake and Ca^2+^-stimulated ATPase activities were depressed by PMLC, unlike PPLC [68,69,71], the possibility arises that PPLC may antagonize the effects of PMLC on SR preparations. Specificity of this interaction on the SR membrane was also tested by measuring Ca^2+^-stimulated ATPase activity of the MF preparation. For this purpose, both SR and MF preparations from control hearts were incubated with 50 μM PPLC for 5 min, and then 50 μM PMLC was added for 5 min before measuring SR Ca^2+^-uptake and Ca^2+^-stimulated ATPase activity as well as MF Ca^2+^-stimulated ATPase activity. The concentrations of PPLC and PMLC were selected on the basis of earlier studies in our laboratory [61,71].

### 4.6. Myocardial and Plasma Biochemical Analysis

Ventricular carnitine and total long-chain acylcarnitine content were measured according to the methods described elsewhere [66,76]. Plasma concentrations of insulin, glucose, free fatty acids, triglycerides and cholesterol were measured by methods used previously [53,74,75].

### 4.7. Statistical Analysis

Data values for control, untreated diabetic and PPLC-treated diabetic animals are expressed as the mean ± SE of 4 experiments in each group. These data were evaluated statistically by using unpaired Student’s “t” test for control vs. diabetic as well as diabetic vs. PPLC-treated diabetic unless indicated in the figure legend. A *p* value < 0.05 was considered significant.

## 5. Conclusions

It is now well known that diabetic cardiomyopathy, cardiac dysfunction and subcellular abnormalities (such as mitochondria, SL, SR and MF defects) occur in chronic diabetes. These alterations in the diabetic heart have been suggested to be associated with multifactorial mechanisms, including hormonal imbalance, oxidative stress, inflammation, Ca^2+^-handling abnormalities and metabolic defects. In this study, we demonstrated that the treatment of diabetic animals with a metabolic intervention (PPLC; a short-chain acylcarnitine) improved cardiac function and lowered the elevated levels of plasma free fatty acids and triglycerides without affecting the elevated level of plasma glucose or the depressed level of plasma insulin. In addition, PPLC treatment prevented the occurrence of a decrease in carnitine content and an increase in total long-chain acylcarnitine content in the diabetic heart. These observations are consistent with the view that partial prevention of cardiac dysfunction in diabetic cardiomyopathy may be a consequence of some metabolic abnormalities in the heart. In addition, this study revealed that PPLC therapy attenuated diabetes-induced alterations in SR Ca^2+^-uptake and Ca^2+^-stimulated ATPase activities without affecting the depressed activities of MF Ca^2+^-stimulated or Mg^2+^-ATPase activities, when measured at different times of incubation as well as at various concentrations of Ca^2+^. PPLC therapy was also observed to attenuate changes in SR ATP-dependent Ca^2+^-binding without affecting the reduced myosin ATPase activities. These observations not only confirm but also extend our previous observations. Furthermore, the differential effects of PPLC therapy on SR and MF activities in diabetic cardiomyopathy may be due to differences in the antagonist effects of PPLC on long-chain acylcarnitine levels, which are increased in the diabetic heart but, unlike SR membranes, do not affect contractile proteins. This view is supported by the fact that PMLC (a long-chain acylcarnitine) was observed to depress SR Ca^2+^-transport activities without any effect on MF ATPase activity under in vitro conditions. Furthermore, PPLC was found to antagonize the PMLC-induced depression in SR Ca^2+^-transport activities. Depression in the elevated levels of long-chain acylcarnitine due to PPLC treatment could also be seen to explain the beneficial effects of PPLC on depressed SR Ca^2+^-uptake and Ca^2+^-stimulated ATPase activities in the diabetic heart. It is therefore suggested that PPLC therapy may improve cardiac function in diabetic cardiomyopathy by preventing Ca^2+^-handling abnormalities as a consequence of changes in SR Ca^2+^-transport function without affecting MF defects.

## Figures and Tables

**Table 1:** **Plasma glucose, insulin and cholesterol levels as well as some hemodynamic parameters of diabetic rats with or without propionyl L-carnitine (PPLC)** **treatment.**

	Control	Diabetic	PPLC-Treated Diabetic
Body weight (g)	426 ± 12	264 ± 7 *	276 ± 11
Ventricular weight (g)	1.3 ± 0.05	0.8 ± 0.03 *	0.9 ± 0.05
Ventricular weight/body weight ratio (mg/g)	2.11 ± 0.06	3.03 ± 0.08 *	3.26 ± 0.09
Systolic blood pressure (mm Hg)	118 ± 7.6	141 ± 5.7 *	112 ± 6.4 ^†^
Heart rate (beats/min)	420 ± 17	326 ± 21 *	358 ± 24
Plasma insulin (μU/ml)	28.6 ± 1.5	10.4 ± 1.3 *	12.5 ± 1.2
Plasma glucose (mg/100 mL)	148 ± 14	674 ± 21 *	635 ± 28
Plasma cholesterol (nmol/L)	1.55 ± 0.27	3.43 ± 0.19 *	2.83 ± 0.25

Three days after inducing diabetes with streptozotocin (65 mg/kg), the diabetic animals were treated with or without PPLC (250 mg/kg/day) for 8 weeks. Values are mean ± SE for 4 animals in each group. * *p* < 0.05 vs. control group; ^†^ <0.05 vs. diabetic group.

**Table 2:** **Ca^2+^-uptake activity at different times of incubation of cardiac sarcoplasmic reticulum from diabetic rats with or without propionyl** **L-carnitine (PPLC) treatment.**

Time of Incubation	Ca^2+^-Uptake (nmol Ca^2+^/mg Protein)		
	Control	Diabetic	PPLC-Treated Diabetic
1 min	32.6 ± 2.1	12.4 ± 1.3 *	20.2 ± 2.7 ^†^
2 min	60.4 ± 3.9	25.6 ± 2.9 *	42.1 ± 3.8 ^†^
5 min	91.6 ± 5.2	56.4 ± 3.8 *	73.5 ± 4.6 ^†^
10 min	138 ± 5.7	80.0 ± 4.3 *	108.6 ± 5.2 ^†^
20 min	150 ± 5.8	94.7 ± 4.4 *	127.4 ± 5.5 ^†^

Three days after inducing diabetes, the diabetic animals were treated with or without PPLC (250 mg/kg/day) for 8 weeks. The sarcoplasmic reticulum was isolated from the ventricular tissue from 2 hearts for each preparation. Ca^2+^-uptake was determined in the presence of 10 μM Ca^2+^. Values are mean ± SE of 4 preparations in each group. * *p* < 0.05 vs. control group; ^†^ *p* < 0.05 vs. diabetic group.

**Table 3:** **ATP-dependent Ca^2+^-binding and ATPase activities of Cardiac sarcoplasmic reticulum from diabetic rats with or without propionyl** **L-carnitine (PPLC) treatment.**

	Control	Diabetic	PPLC-Treated Diabetic
ATP-independent Ca^2+^-binding (nmol Ca^2+^/mg/5 min)	5.0 ± 0.02	4.8 ± 0.03	5.1 ± 0.03
ATP-dependent Ca^2+^-binding (nmol Ca^2+^/mg/5 min)	21.6 ± 1.30	10.4 ± 0.85 *	16.2 ± 0.94 ^†^
Mg^2+^-ATPase activity (μmol Pi/mg/5 min)	12.4 ± 0.84	11.2 ± 0.96	10.8 ± 0.92
Ca^2+^-stimulated ATPase activity (μmol Pi/mg/5 min)	0.96 ± 0.03	0.65 ± 0.03 *	0.82 ± 0.02 ^†^

Three days after inducing diabetes, the diabetic animals were treated with or without 250 mg/kg/day PPLC for 8 weeks. The sarcoplasmic reticulum was isolated from the ventricular tissue from 2 hearts for each preparation. Ca^2+^- binding in the absence or presence of ATP as well as ATPase activities were determined. The concentration of Ca^2+^ was 10 μM. Values are mean ± SE of 3 preparations in each group. * *p* < 0.05 vs. control group; ^†^ *p* < 0.05 vs. diabetic group.

**Table 4:** **ATP** **activities of myofibrils and myosin from diabetic rats with or without propionyl L-carnitine (PPLC) treatment.**

	Control	Diabetic	PPLC-Treated Diabetic
A. Myofibrillar ATPase Activity:			
Mg^2+^-ATPase activity (nmol Pi/mg/5 min)	264 ± 10	176 ± 12 *	188 ± 14
Ca^2+^-stimulated ATPase activity (nmol Pi/mg/5 min)	972 ± 24	586 ± 19 *	632 ± 18
B. Myosin ATPase Activity:			
Ca^2+^-ATPase activity (nmol Pi/mg/5 min)	376 ± 22	276 ± 15 *	284 ± 14
Actin-stimulated ATPase activity (nmol Pi/mg/5 min)	254 ± 14	182 ± 13 *	192 ± 17

Three days after the induction of diabetes, the diabetic animals were treated with or without PPLC (250 mg/kg/day) for 8 weeks. Myofibrils and myosin were isolated from the ventricular muscle from 2 hearts for each preparation, and their ATPase activities were determined. The concentration of Ca^2+^ was 10 μM. Values are means ± SE of 4 preparations in each group. * *p* < 0.05 vs. control.

**Table 5:** **Ca^2+^-stimulated ATPase activities at different times of incubation of myofibrils from diabetic animals with or without propionyl** **L-carnitine (PPLC) treatment.**

Time of Incubation	Ca^2+^-Stimulated ATPase Activity (nmol Pi/mg/5 min)		
	Control	Diabetic	PPLC-Treated Diabetic
1 min	266 ± 11	148 ± 16 *	172 ± 12
2 min	484 ± 18	306 ± 15 *	352 ± 17
5 min	1128 ± 22	690 ± 24 *	756 ± 29
10 min	1678 ± 25	1080 ± 31 *	1147 ± 31
20 min	1892 ± 33	1374 ± 30 *	1446 ± 27

Three days after the induction of diabetes, the diabetic rats were treated with or without PPLC (250 mg/kg/day) for 8 weeks. Myofibrils were isolated from the ventricular muscle from 2 hearts for each preparation. Ca^2+^-stimulated ATPase activity was determined at different times of incubation. The concentration of Ca^2+^ was 10 μM. Values are means ± SE of 4 preparations in each group. * *p* < 0.05 vs. control group.

**Table 6:** **Effect of palmitoyl L-Carnitine (PMLC) or propionyl L-carnitine (PPLC) plus PMLC in control rat heart myofibrils (MFs) at different concentrations** **of Ca^2+^.**

Concentrations of Ca^2+^	Ca^2+^-Stimulated ATPase Activity (nmol Pi/mg/5 min)		
	Control	50 μM PMLC	50 μM PPLC + 50 μM PMLC
0.1 μM	252 ± 24	264 ± 1.8	246 ± 22
0.5 μM	516 ± 32	504 ± 23	524 ± 31
1.0 μM	694 ± 28	676 ± 31	668 ± 30
5.0 μM	786 ± 36	780 ± 35	776 ± 28
10 μM	896 ± 32	878 ± 32	870 ± 32

MFs were isolated from the ventricular muscle from 2 hearts in each preparation. MFs were treated in vitro with or without PPLC for 5 min before adding PMLC in the incubation medium for 5 min, and then ATPase activities were determined. Values are means ± SE for 3 preparations in each group.

## Data Availability

The data that support the findings of this study are available within this article and from the corresponding author upon reasonable individual request.

## Abbreviations

PPLC, propionyl L-carnitine; PMLC, palmitoyl L-carnitine; SR, sarcoplasmic reticulum; MF, myofibril; SL, sarcolemma.
